# Benchmarking large language models for genomic knowledge with GeneTuring

**DOI:** 10.1101/2023.03.11.532238

**Published:** 2025-01-05

**Authors:** Wenpin Hou, Xinyi Shang, Zhicheng Ji

**Affiliations:** 1 Department of Biostatistics, The Mailman School of Public Health, Columbia University, New York City, NY, USA; 2 Department of Biostatistics and Bioinformatics, Duke University School of Medicine, Durham, NC, USA.

**Keywords:** Large language model, Genomics, Benchmark, Knowledge base

## Abstract

Large language models have demonstrated great potential in biomedical research. However, their ability to serve as a knowledge base for genomic research remains unknown. We developed GeneTuring, a comprehensive Q&A database containing 1,200 questions in genomics, and manually scored 25,200 answers provided by six GPT models, including GPT-4o, Claude 3.5, and Gemini Advanced. GPT-4o with web access showed the best overall performance and excelled in most tasks. However, it still failed to correctly answer all questions and may not be fully reliable for providing answers to genomic inquiries.

## Introductions

Large language models (LLMs), such as GPT-3.5^1^, GPT-4^2^, Gemini^3^, and Claude^4^, are advanced models trained on massive datasets, capable of producing text that closely resembles human speech. LLMs excel in various tasks, such as answering questions^2^, generating programming code^5^, and analyzing images^6^. Recent studies have also highlighted their strong capabilities in genomic research. For instance, in single-cell RNA-seq data, GPT-4 can produce cell type annotations that are highly consistent with those provided by human experts, using only marker gene information as input^7^. Additionally, gene embeddings generated by GPT-3.5 can be utilized to create single-cell embeddings for various downstream analyses^8^.

These studies suggest that LLMs possess knowledge in the field of genomics and have the potential to serve as a knowledge base for genomic research. Such an LLM-based genomic knowledge base could significantly benefit genomic research by reducing the time required to locate and retrieve reliable information, a process that is often time-consuming for interdisciplinary researchers with limited genomic expertise. Moreover, the advanced reasoning and analytical capabilities of modern LLMs enable efficient synthesis of information from diverse sources. However, whether LLMs can reliably serve as genomic knowledge bases has not been systematically studied and remains poorly understood.

Benchmark datasets are essential for comparing and assessing the ability of LLMs to perform specific tasks. For example, MMLU (Massive Multitask Language Understanding)^9^ is a widely used benchmark dataset for evaluating LLMs’ interdisciplinary knowledge, while HumanEval^10^ assesses their ability to generate programming code. These benchmark datasets provide a standardized framework for comparing performance across different LLMs and tracking model evolution over time. They have been pivotal in identifying the weaknesses of existing models and guiding future development toward more advanced LLMs. However, existing benchmark datasets do not cover genomics, and a benchmark for genomic knowledge is still lacking.

To this end, we developed GeneTuring, a comprehensive question-and-answer (Q&A) database, to benchmark the performance of LLMs in genomics. GeneTuring encompasses various aspects of genomic research, such as the genomic locations of genes and SNPs, as well as the functions of genes. We evaluated the performance of six LLMs on GeneTuring, including BioGPT^11^, BioMedLM^12^, GPT-3.5^1^, GPT-4o^13^, Gemini Advanced^3^, and Claude 3.5^4^. Among these, GPT-4o, Gemini Advanced, and Claude 3.5 are contemporary LLMs widely recognized for their strong performance across various tasks.

Our analysis revealed significant variation in the accuracy of genomic knowledge-based question answering across the LLMs. Moreover, we observed that performance could be further enhanced when LLMs have access to web browsing capabilities. However, even the best-performing LLM completely failed in certain tasks and were unable to answer all questions correctly in others, despite the likelihood that the genomic knowledge was included in their training corpora. These findings suggest that current LLMs fall short of serving as reliable knowledge bases for genomic research due to issues such as AI hallucination^14,15^. More advanced LLMs will be required to address these limitations in the future.

## Results

**Figure 1.**
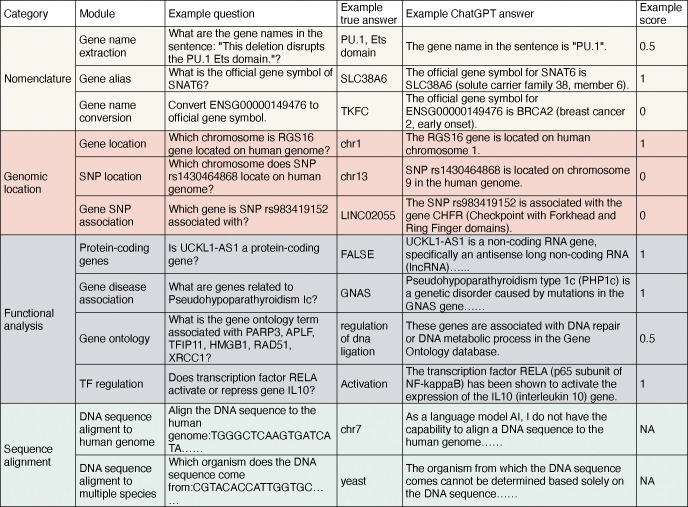
A table showing the categories and names of the twelve modules, example pairs of questions and true answers, example answers from ChatGPT, and scores of the example answers.

GeneTuring consists of twelve modules containing a total of 1200 question-answer pairs, which are grouped into four categories: nomenclature, genomic location, functional analysis, and sequence alignment. These twelve modules represent tasks commonly encountered in genomics research. Figure 1 illustrates the categories and names of the twelve modules, along with example question-answer pairs, sample responses from GPT-3.5, and the corresponding scores of these responses. To ensure compatibility with models based on GPT-2, the same questions were reformulated as sentence completion tasks (Methods).

We used GeneTuring to evaluate six LLMs: BioGPT^11^, BioMedLM^12^, GPT-3.5^1^, GPT-4o^13^, Gemini Advanced^3^, and Claude 3.5^4^. GPT-3.5 and GPT-4o are commercial models developed by OpenAI, while Gemini Advanced is a commercial model developed by Google. Claude 3.5, another commercial model, was developed by Anthropic. BioGPT and BioMedLM are based on OpenAI’s GPT-2 architecture but are specifically trained on biomedical literature. We accessed all these models via the API, where none of them have access to web content. To evaluate whether access to web content impacts performance, we also tested GPT-4o using the web browser version, which enables web browsing capabilities.

For GPT-3.5, GPT-4o, Gemini Advanced, and Claude 3.5, each question was presented three times, and the three responses were recorded. For BioGPT and BioMedLM, each question was presented once, and the top three answers returned by the model were recorded. For each of the 25,200 responses, we manually evaluated whether the model correctly understood the question, acknowledged its inability to answer the question (a concept we term “incapacity awareness”), or failed to provide a relevant answer. If a relevant answer was provided, a numeric score between 0 and 1 was assigned based on a predefined scoring mechanism (Methods). All Q&A pairs used in the evaluation, responses from the LLMs, and the corresponding scores are provided in Supplementary Table 1.

**Figure 2.**
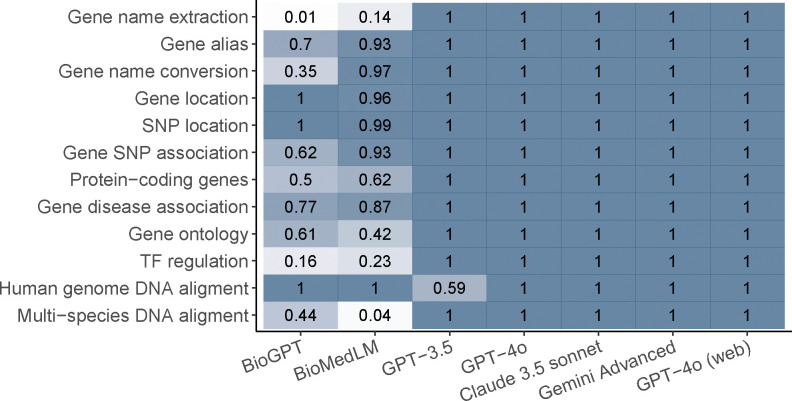
The proportion of questions that are correctly understood by each method for each module.

We first examined whether the LLMs can understand the questions and generate relevant answers (Figure 2). GPT-4o, Claude 3.5, and Gemini Advanced provide relevant answers to all questions, while GPT-3.5 does so for almost all questions. In contrast, models based on GPT-2 (BioGPT and BioMedLM) struggle to understand questions in many tasks. The inferior performance of GPT-2-based models is likely due to their reliance on a sentence completion mechanism or their smaller model capacities. Since understanding the question is a prerequisite for providing correct answers, these results highlight the importance of using advanced model architectures that excel in natural language understanding.

**Figure 3.**
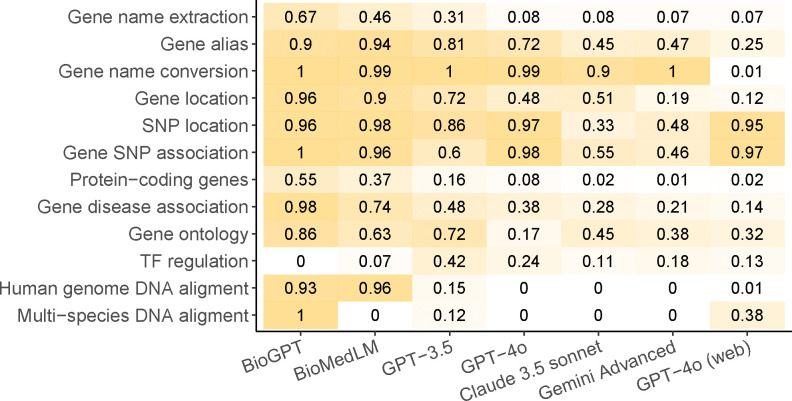
The proportion of answers with zero scores (AI hallucinations), calculated only for questions that are correctly understood.

We next examined how likely LLMs are to generate false answers, given that the question was correctly understood. Figure 3 shows the proportion of questions that receive a score of zero for their answers. In these cases, LLMs provide inaccurate information despite the seemingly confident claims made by these models, a phenomenon recognized as AI hallucination. AI hallucination is widely observed across different tasks and LLMs, even in advanced models such as GPT-4o, Claude 3.5, and Gemini Advanced. For example, GPT-4o produces false results in almost all cases in the two tasks related to SNP locations, and nearly all models exhibit severe AI hallucination in the task of gene name conversion.

Surprisingly, GPT-4o with access to web browsing drastically improves the accuracy of results in the task of gene name conversion, generating errors in only 1% of cases. In comparison, GPT-4o without online access generates errors in 99% of cases. Similarly, the error rate drops substantially in the tasks of gene alias and gene location when GPT-4o has access to web browsing. However, online access is not a universal solution for all tasks. For instance, the error rate remains largely unchanged in the two SNP-related tasks for GPT-4o, regardless of whether it has online access. A potential explanation is that information on gene names and gene IDs is readily available online, whereas most SNPs are rare variants that are not discussed in any literature or online resource. Thus, LLMs can only benefit from access to online resources when the relevant knowledge is available online.

**Figure 4.**
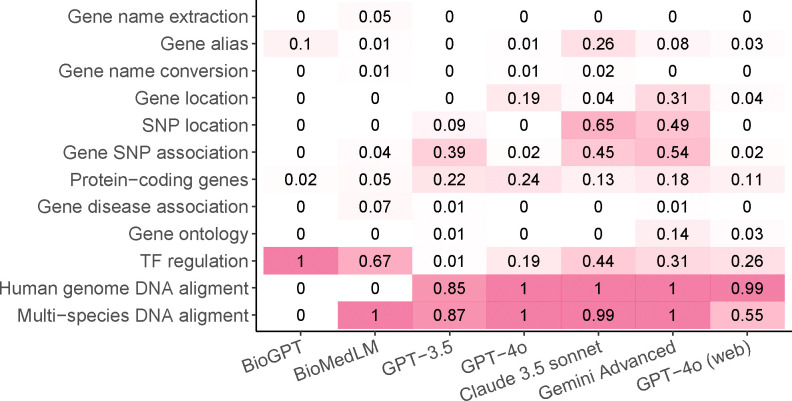
The proportion of questions where the model acknowledges its incapacity in answering the question (incapacity awareness), calculated only for questions that are correctly understood.

We further investigated why AI hallucination is less severe in certain cases. We found that LLMs are sometimes able to recognize and report their inability to answer a question without providing a definite response, a phenomenon we refer to as “incapacity awareness.” For example, in one of the multi-species DNA alignment tasks, Gemini Advanced responded, “Unfortunately, determining the exact organism from this short DNA sequence alone is not possible.” Figure 4 shows the proportion of cases where LLMs report their incapacity to answer, given that the question is properly understood. This proportion is particularly high in the two tasks related to DNA sequence alignment, explaining the relatively low occurrence of AI hallucinations in these tasks. Conversely, as expected, the proportion of incapacity awareness is low in tasks such as gene name conversion, where AI hallucination is more prevalent.

We argue that incapacity awareness is crucial for addressing AI hallucination, particularly in scientific research areas such as genomics. Each version of an LLM is trained on a finite dataset, and inevitably, there will be questions that fall outside the scope of its training data, making it unlikely for the model to provide correct answers. Admitting these limitations alerts users to seek alternative approaches. In contrast, when LLMs generate random or false answers, users are often unaware of the inaccuracies, which can lead to further issues, such as wasted time and resources on validation experiments for incorrect targets.

**Figure 5.**
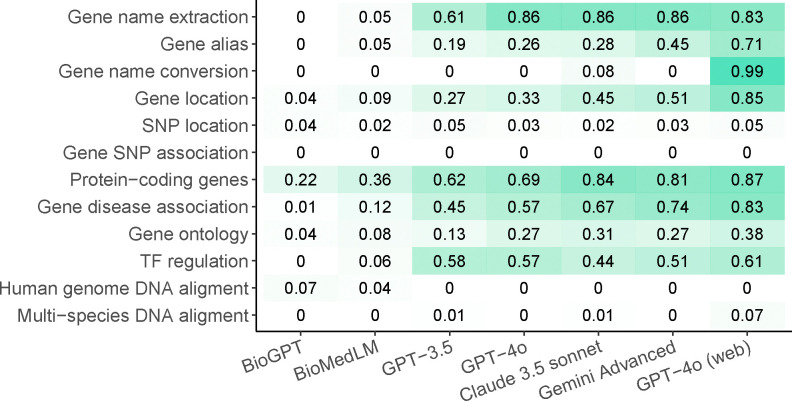
The overall score, which is the average of scores across all answers in that module.

Finally, we report the overall score, calculated as the average score of all answers within each module for each LLM (Figure 5). A score of zero is assigned when a question is not correctly understood or when the model acknowledges its incapacity. GPT-4o with web access achieves the best overall performance and performs reasonably well across almost all tasks unrelated to SNPs or sequence analysis. A potential reason why GPT-4o does not excel in all tasks is that SNP and DNA sequence information are unavailable in the training data and not accessible online. However, GPT-4 fails to achieve 100% accuracy in any single task, suggesting that even the best-performing LLM cannot serve as a fully reliable knowledge base. When online access is not available, GPT-4o, Claude 3.5, and Gemini Advanced show comparable performance, with Gemini Advanced achieving the best results among the three. However, their performances are substantially worse than GPT-4o with online access in tasks such as gene alias, gene name conversion, and gene location. BioGPT, BioMedLM, and GPT-3.5, which are based on earlier LLM architectures, exhibit the poorest overall performance.

## Conclusions

We developed GeneTuring, a Q&A benchmarking database designed to evaluate the performance of LLMs in answering questions related to genomics. We found that performance varies substantially across different LLMs and that AI hallucination is prevalent in all scenarios. Despite being the best-performing LLM, GPT-4o with web access still falls short as a reliable genomic knowledge base and fails to correctly answer all questions in any of the twelve modules. Therefore, precautions should be taken when using LLMs to answer genomics-related questions.

## Methods

### GeneTuring compilation and scoring criteria

GeneTuring comprises 12 modules, each containing 100 pairs of questions and answers. Additionally, GeneTuring converts each question-and-answer pair into a sentence completion task with the same meaning to accommodate models built on GPT-2 architectures. The details of how the 12 modules were compiled and scored numerically are discussed below. Notably, only relevant and definitive answers from LLMs were scored numerically. Outputs from LLMs were not scored numerically if they failed to directly answer the questions or acknowledged their limitations in doing so.

#### Gene name extraction

This module evaluates the ability of LLMs to extract gene and gene product names from a given sentence. We downloaded the test set for the BioCreative II Challenge Task 1A: Gene Mention Tagging (BC2GM) from the BioCreative website^16^. The test set consists of 5,000 pairs of sentences and the corresponding names of genes and gene products mentioned in those sentences. From this set, 100 pairs were randomly selected.

To create question-answering tasks, a question was generated by appending the phrase “What are the gene and protein names in the sentence: “ before the BC2GM sentence and adding a question mark at the end. For sentence completion tasks, a prompt was created by appending the phrase “The gene and protein names in the sentence “ before the BC2GM sentence and adding “ is” at the end. The gene and gene product names provided by the original BC2GM task served as the gold standard answers. Each sentence may contain zero, one, or multiple gene and gene product names.

A Jaccard index was used to evaluate the performance of the answers provided by an LLM. Let A represent the set of gene and gene product names identified by the LLM, and B represent the gold standard set of gene and gene product names. The Jaccard index is calculated as $\frac{\left|A\cap B\right|}{\left|A\cup B\right|}$, where |.| denotes the cardinality of a set. If both the gold standard and the LLM report no gene or gene product names (A=∅ and B=∅), the Jaccard index is set to 1.

#### Gene alias

This module evaluates the ability of LLMs to identify the official gene symbol for a given gene alias. The information on official gene names and their aliases for human protein-coding genes was downloaded from the NCBI website^17^. A total of 100 genes with at least one alias were randomly selected. For each gene, one alias was randomly chosen to generate the question.

To create question-answering tasks, a question was generated by appending “What is the official gene symbol of “ before the alias and adding a question mark after it. For sentence completion tasks, a prompt was created by appending “The official gene symbol of gene “ before the alias and adding “ is” after it. The official gene symbol was used as the gold standard.

Similar to the previous section, a Jaccard index was used to compare the official gene symbols provided by an LLM with the gold standard and to assign a score. The Jaccard index was employed because LLMs may provide multiple official gene symbols as answers to some questions.

#### Gene name conversion

This module evaluates the ability of LLMs to convert Ensembl gene names into gene symbols. The gene annotation GTF file for the human GRCh38 genome was downloaded from the GENCODE website^18^. A total of 100 protein-coding genes were randomly selected, and the Ensembl gene name and corresponding gene symbol for each gene were recorded.

To create question-answering tasks, a question was generated by appending “Convert “ before the Ensembl gene name and “ to official gene symbol.” after it. For sentence completion tasks, a prompt was created by appending “The official gene symbol of “ before the Ensembl gene name and “ is” after it. The gene symbol was used as the gold standard.

A score of 1 was assigned if the gene symbol provided by the LLM matched the gold standard, and a score of 0 was assigned if it did not.

#### Gene location

This module evaluates the ability of LLMs to determine which chromosome a gene is located on. The gene annotation GTF file for the human GRCh38 genome was downloaded from the GENCODE website^18^. Genes with multiple locations on the genome were excluded. A total of 100 genes were randomly selected, and for each gene, its gene symbol and the chromosome name it is located on were recorded.

To create question-answering tasks, a question was generated by appending “Which chromosome is “ before the gene symbol and “ gene located on the human genome?” after it. For sentence completion tasks, a prompt was generated by appending “ gene is located on human genome chromosome” after the gene symbol. The chromosome name was used as the gold standard.

A score of 1 was assigned if the chromosome name provided by the LLM matched the gold standard, and a score of 0 was assigned if it did not.

#### SNP location

This module evaluates the ability of LLMs to identify which chromosome a single nucleotide polymorphism (SNP) is located on. The SNP locations were downloaded from the NCBI website^17^. A total of 100 SNPs were randomly selected. For each SNP, its symbol and the name of the chromosome it is located on were recorded.

To create question-answering tasks, a question was generated by appending “Which chromosome does SNP “ before the SNP symbol and “ locate on the human genome?” after it. For sentence completion tasks, a prompt was generated by appending “SNP “ before the SNP symbol and “ is located on human genome chromosome” after it. The chromosome name was used as the gold standard.

A score of 1 was assigned if the chromosome name provided by the LLM matched the gold standard, and a score of 0 was assigned if it did not.

#### Gene SNP association

This module evaluates the ability of LLMs to identify which gene a single nucleotide polymorphism (SNP) is associated with. SNP-gene association information was downloaded from the NCBI website^17^. SNPs associated with zero or more than one gene were excluded. A total of 100 SNPs were randomly selected, and for each SNP, its symbol and the associated gene symbol were recorded.

To create question-answering tasks, a question was generated by appending “Which gene is SNP “ before the SNP symbol and “ associated with?” after it. For sentence completion tasks, a prompt was generated by appending “The name of the gene associated with SNP “ before the SNP symbol and “ is” after it. The gene symbol was used as the gold standard.

A score of 1 was assigned if the gene symbol provided by the LLM matched the gold standard, and a score of 0 was assigned if it did not.

#### Protein-coding genes

This module evaluates the ability of LLMs to determine whether a gene codes for a protein. Gene information was downloaded from the NCBI website^17^. A total of 100 human genes, including protein-coding genes, non-coding RNA (ncRNA) genes, and pseudogenes, were randomly selected. For each gene, its symbol and gene type were recorded.

To create question-answering tasks, a question was generated by appending “Is “ before the gene symbol and “ a protein-coding gene?” after it. For sentence completion tasks, a prompt was generated by appending “Regarding whether the gene codes for a protein, “ before the gene symbol and “ is” after it. A binary value indicating whether the gene is a protein-coding gene was used as the gold standard.

A score of 1 was assigned if the LLM’s response matched the gold standard in determining whether the gene is a protein-coding gene, and a score of 0 was assigned if it did not.

#### Gene disease association

This module evaluates the ability of LLMs to identify genes associated with specific diseases. Gene-disease association information was downloaded from the OMIM website^19^. A total of 100 diseases were randomly selected, and all genes associated with each disease were recorded.

To create question-answering tasks, a question was generated by appending “What are the genes related to “ before the disease name and adding a question mark after it. For sentence completion tasks, a prompt was generated by appending “The name of the gene related to “ before the disease name and “ is” after it. The set of genes associated with each disease was used as the gold standard.

The answer provided by an LLM was scored based on the proportion of gold standard genes mentioned in the response.

#### Gene ontology

This module evaluates the ability of LLMs to identify gene ontology (GO) terms enriched in a set of genes. GO information for biological processes was downloaded from MSigDB^20^. A total of 100 GO terms were randomly selected, and all genes associated with each GO term were recorded.

To create question-answering tasks, a question was generated by appending “What is the enriched gene ontology term associated with “ before the list of genes and adding a question mark after the list of genes. For sentence completion tasks, a prompt was generated by appending “The enriched gene ontology term associated with “ before the list of genes and “ is” after the list of genes. The name of the GO term was used as the gold standard.

A score of 1 was assigned if one of the GO terms provided by an LLM fully matched the gold standard. A score of 0.5 was assigned if one of the GO terms partially matched the gold standard. A score of 0 was assigned if none of the GO terms provided by the LLM fully or partially matched the gold standard.

#### TF regulation

This module tests the ability of LLMs to determine whether a transcription factor activates or represses the expression of a gene. Information on transcription factors regulating genes in humans was obtained from the Trrust database^21^. A total of 100 pairs of transcription factors and genes with known activation or repression relationships were randomly selected.

To create question-answering tasks, questions were generated by combining the phrases: “Does transcription factor,” the name of the transcription factor, “activate or repress gene,” the name of the gene, and “?” into a single sentence. Similarly, to create sentence completion tasks, sentences were generated by combining: “The regulatory relationship between transcription factor,” the name of the transcription factor, “and gene,” the name of the gene, and “is” into a single prompt. A binary value indicating whether the transcription factor activates the gene served as the gold standard.

A score of 1 was assigned if the LLM’s response agreed with the gold standard in identifying whether the relationship was activation or repression, while a score of 0 was assigned if they disagreed.

#### DNA sequence aligment to human genome

This module evaluates the ability of LLMs to identify the chromosome to which a DNA sequence aligns in the human genome. DNA sequence information for the human genome was obtained from the Bioconductor package BSgenome.Hsapiens.UCSC.hg38^22^. Only autosomes and sex chromosomes were retained, and regions containing “N” in the DNA sequence were excluded.

To select a genomic region, a chromosome was randomly chosen, along with a starting position within the range of the selected chromosome and a length between 100 and 150 base pairs. The corresponding DNA sequence was extracted based on the selected chromosome, starting position, and length. This process was repeated 100 times to generate 100 DNA sequences and their associated chromosome names.

For question-answering tasks, questions were generated by appending “Align the DNA sequence to the human genome:” before the DNA sequence. For sentence completion tasks, questions were created by appending “The DNA sequence” before the DNA sequence and “is on the human genome chromosome” after the DNA sequence. The chromosome name was used as the gold standard.

A score of 1 was assigned if the chromosome name provided by the LLM matched the gold standard, and a score of 0 was assigned if it did not.

#### DNA sequence aligment to multiple species

This module evaluates the ability of LLMs to identify the species from which a DNA sequence originates. DNA sequence information for human, mouse, rat, chicken, zebrafish, worm, and yeast genomes was obtained from the Bioconductor^22^ packages: BSgenome.Hsapiens.UCSC.hg38, BSgenome.Mmusculus.UCSC.mm10, BSgenome.Rnorvegicus.UCSC.rn5, BSgenome.Ggallus.UCSC.galGal6, BSgenome.Drerio.UCSC.danRer11, BSgenome.Celegans.UCSC.ce11, and BSgenome.Scerevisiae.UCSC. Only autosomes and sex chromosomes were retained, and regions containing “N” in the DNA sequence were excluded.

To select a genomic region, one of the seven species was randomly chosen, followed by the random selection of a chromosome within the chosen species, a starting position within the range of the selected chromosome, and a length between 100 and 150 base pairs. The corresponding DNA sequence was extracted based on the selected species, chromosome, starting position, and length. This process was repeated 100 times to generate 100 DNA sequences along with their respective species names.

For question-answering tasks, questions were created by appending “Which organism does the DNA sequence come from:” before the DNA sequence. For sentence completion tasks, questions were created by appending “The name of the species where the DNA sequence” before the DNA sequence and “comes from is” after the DNA sequence. The species name served as the gold standard.

A score of 1 was assigned if the species name provided by the LLM matched exactly with the gold standard. A score of 0.5 was assigned if the LLM provided a correct superset of the gold standard but did not match it exactly. A score of 0 was assigned if the LLM’s answer was neither a superset nor an exact match with the gold standard.

### LLMs

The BioGPT and BioMedLM models were accessed using the interface provided by Hugging Face^23^. GPT-3.5 (gpt-3.5-turbo-0125) was accessed via the API provided by OpenAI. Claude 3.5 (claude-3–5-sonnet-20240620) was accessed via the API provided by Anthropic. GPT-4o without online access (gpt-4o-2024–05-13) was accessed via the API provided by OpenAI. Gemini Advanced was accessed through its web browser version (https://gemini.google/advanced/). GPT-4o with online access was accessed through its web browser version (https://chat.openai.com/).

## Key points

- We developed GeneTuring, a comprehensive Q&A database designed to facilitate genomic knowledge.
- We evaluated six LLMs using GeneTuring and found that GPT-4o with web access demonstrated the best performance.
- While GPT-4o excels in most tasks, it fails to provide accurate answers for all tasks.
- These findings suggest that current LLMs still fall short of being a reliable genomic knowledge base.
