# Supplementary material for: Benchmarking large language models for genomic knowledge with GeneTuring
Source: bioRxiv. 2025 Sep 12:2023.03.11.532238. Originally published 2023 Mar 13. Preprint. [Version 3] doi: 10.1101/2023.03.11.532238 (PMC10054955; doi:10.1101/2023.03.11.532238)
Supplement: Supplement 1 [file media-1.pdf]

Supplementary materials

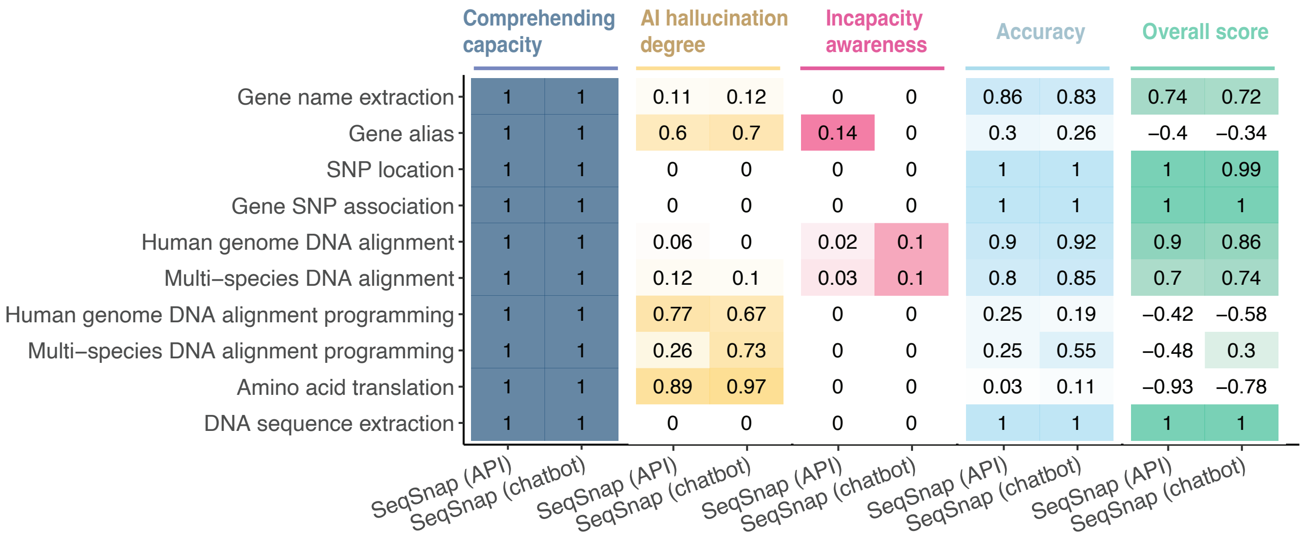

[1] **Figure S1. Performance comparisons of the API and chatbot versions of SeqSnap on GeneTuring modules.**

[1]All questions were assessed for the API version, and the top 10 questions were assessed for the chatbot version due to time constraints.
